# Reproducibility of Frankfort Horizontal Plane on 3D Multi-Planar Reconstructed MR Images

**DOI:** 10.1371/journal.pone.0048281

**Published:** 2012-10-31

**Authors:** Amro Daboul, Christian Schwahn, Grit Schaffner, Silvia Soehnel, Stefanie Samietz, Ahmad Aljaghsi, Mohammad Habes, Katrin Hegenscheid, Ralf Puls, Thomas Klinke, Reiner Biffar

**Affiliations:** 1 Polyclinic of Prosthodontics and Biomaterials, Greifswald University, Greifswald, Germany; 2 Institute of Community Medicine, Greifswald University, Greifswald, Germany; 3 Institute of Diagnostics, Radiology and Neuroradiology, Greifswald University, Greifswald, Germany; University of Toronto, Canada

## Abstract

**Objective:**

The purpose of this study was to determine the accuracy and reliability of Frankfort horizontal plane identification using displays of multi-planar reconstructed MRI images, and propose it as a sufficiently stable and standardized reference plane for craniofacial structures.

**Materials and Methods:**

MRI images of 43 subjects were obtained from the longitudinal population based cohort study SHIP-2 using a T1-weighted 3D sequence. Five examiners independently identified the three landmarks that form FH plane. Intra-examiner reproducibility and inter-examiner reliability, correlation coefficients (ICC), coefficient of variability and Bland-Altman plots were obtained for all landmarks coordinates to assess reproducibility. Intra-examiner reproducibility and inter-examiner reliability in terms of location and plane angulation were also assessed.

**Results:**

Intra- and inter-examiner reliabilities for X, Y and Z coordinates of all three landmarks were excellent with ICC values ranging from 0.914 to 0.998. Differences among examiners were more in X and Z than in Y dimensions. The Bland–Altman analysis demonstrated excellent intra- as well as inter-examiner agreement between examiners in all coordinates for all landmarks. Intra-examiner reproducibility and inter-examiner reliability of the three landmarks in terms of distance showed mean differences between 1.3 to 2.9 mm, Mean differences in plane angulation were between 1.0° to 1.5° among examiners.

**Conclusion:**

This study revealed excellent intra-examiner reproducibility and inter-examiner reliability of Frankfort Horizontal plane through 3D landmark identification in MRI. Sufficiently stable landmark-based reference plane could be used for different treatments and studies.

## Introduction

Since the development of magnetic resonance imaging (MRI), it has been increasingly used for medical diagnosis as an imaging modality with no ionizing radiation. In dentistry, MRI has been used to evaluate the temporomandibular joint, orofacial tissues, implant planning and in longitudinal studies analyzing craniofacial structures. [1]–[7] MRI has been shown to enable accurate and reproducible three-dimensional measurements of the craniofacial skeleton due to the contrast between the bone and the surrounding soft tissue [8], that allows us to evaluate the craniofacial morphology which is difficult to identify with cephalometric radiography.

**Figure 1:**
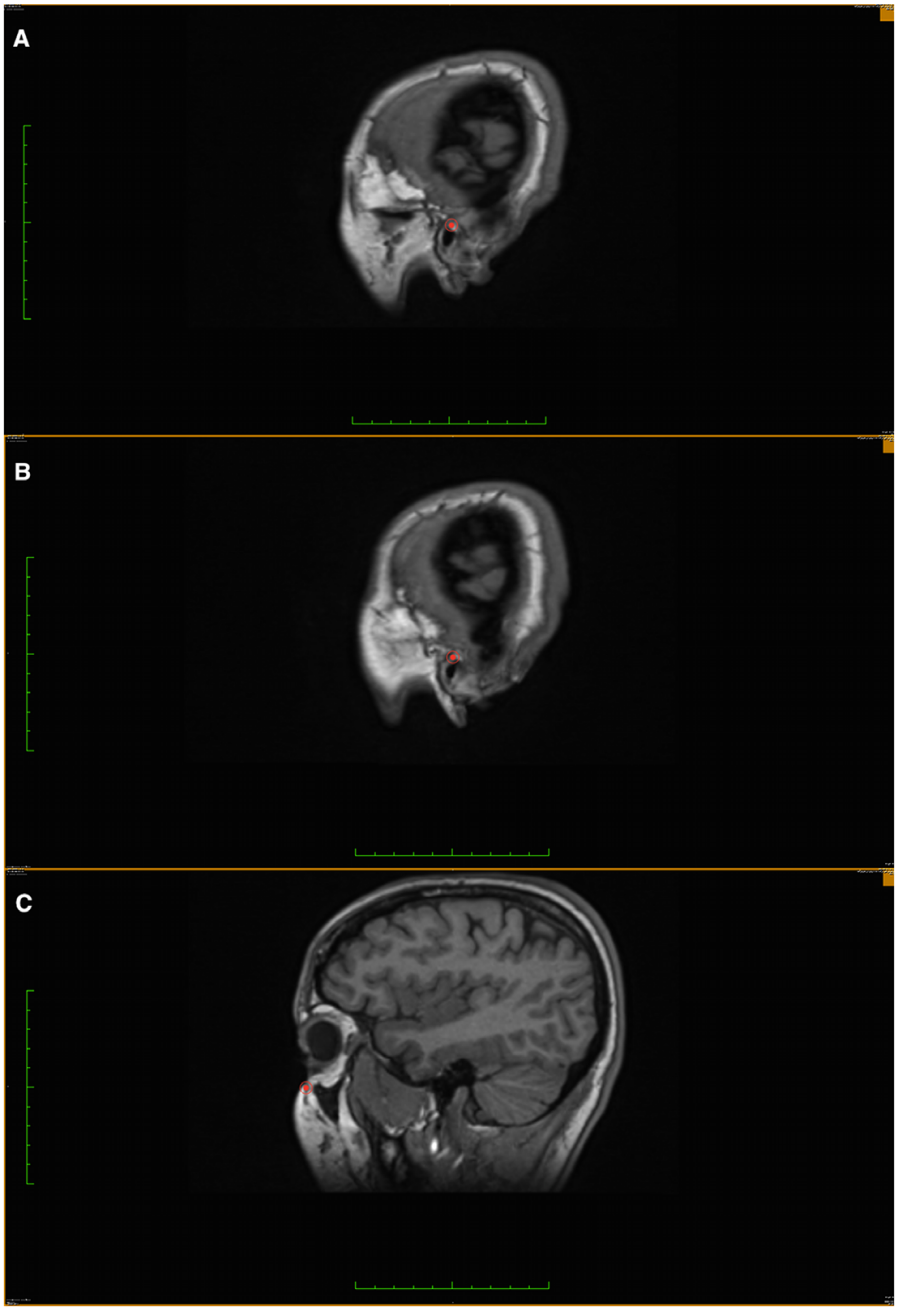
Using coronal view as the orientation source and plotting landmarks on the sagittal view. a) Left Porion. b) Right Porion. c) Left Orbitale.

In three-dimensional (3D) analysis of the craniofacial area, different structures are measured with regard to lines or reference planes. Generally, these reference planes are based on anatomical landmarks that are traditionally used for cephalometric measurements. [9]–[13] Since the validity of craniofacial analysis and measurements depend highly on the accuracy and reliability of the reference plane used, the identification and reproducibility of the reference plane landmarks should be verified in each imaging modality. Furthermore, several factors that contribute to the reliability of landmark identification have to be considered, factors such as scan parameters, quality of images, training level or experience of the examiners, definition of the landmark and anatomic complexity will influence the magnitude of identification errors. [14]–[16].

Previous studies have recommended that every study should include an assessment of reproducibility [17], [18]. While this recommendation is not necessary in clinical studies, it is justified for research work where great precision is required. [19].

Gravely et al [20] concluded that Intra-examiner errors are generally lower than inter-examiner errors in landmarks identified on 2D cepahlometric images, and in a study conducted by Kragskov et al [21], it was suggested that landmarks detection on 3D CT images has less reliability than traditional 2D cephalometric images. Kragskov argued that the reason behind these findings was that distances calculated between points on 2D cephalograms consisted of two coordinates only in comparison to three coordinates for 3D CT images, thus adding an extra deviation. On the contrary, other studies have reported good reproducibility of craniofacial landmarks in 3D CT using phantoms and metallic markers. [22], [23] However, this approach demonstrates the accuracy of the imaging but does not simulate the clinical situation in which precision is influenced by the difficulty in identifying landmarks. [21].

**Figure 2:**
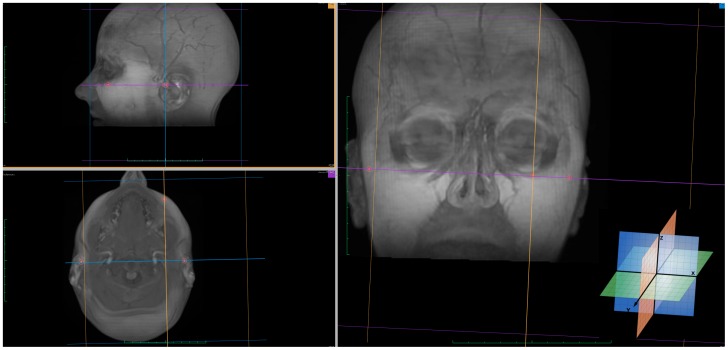
Plotted landmarks on 3D rendered images with MPR view.

The Frankfort Horizontal (FH) was originally introduced at an anthropological conference in Frankfurt, Germany in 1884. It was defined as a plane extending from the left Orbitale to both Porion points. [24] Since then, the plane has been widely recognized as a reference plane for the skull and has proved to be of great value in craniofacial studies and orthodontics. It has been presented in several studies as an adequate cranial base reference and was incorporated in anthropological studies, maxillofacial surgery planning and descriptive communications between clinicians. [25]–[28] However, in many of the previous studies in the craniofacial area, anatomical areas were studied based on FH plane visual estimation rather than landmark identification, FH landmark identification errors were not evaluated on images.

The purposes of the present study were to determine the accuracy and reliability of Frankfort horizontal plane identification using displays of multi- planar reconstructed MRI images, assess the reproducibility of its landmarks and propose it as a standardized reference plane for craniofacial measurements in MRI.

**Figure 3:**
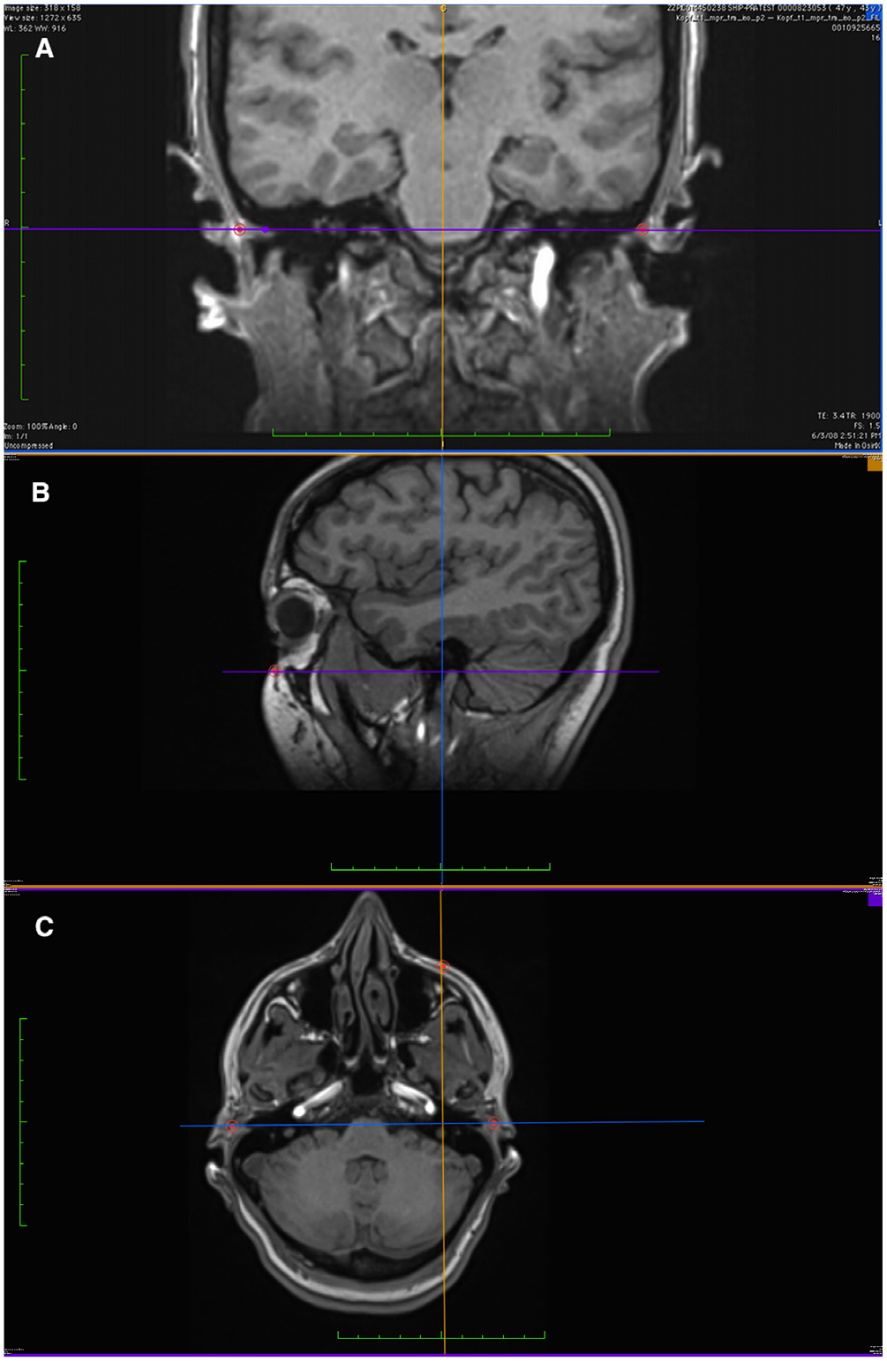
Images were viewed using multi planar reconstruction (MPR) with coronal view as the center of orientation. a) Horizontal and vertical lines were adjusted until the standard trans-porionic axis was observed. b) Balance between axial, coronal and sagittal axis adjusted to connect trans-porionic axis (purple) with Orbitale through a coronal axis (blue). c) Axial view through FH after connecting the 3 axes.

## Materials and Methods

### (a) Subjects and MRI Data Acquisition

This study was carried out on 43 subjects (26 f, 17 m), age between 26–78 years old (with a mean age ± standard deviation of 46.4±12.0) with normal skull shape. Subjects were randomly chosen from the longitudinal population based cohort study SHIP-2 [29], [30] at which the medical ethics committee of University of Greifswald approved the study protocol, and written informed consents were obtained from all the subjects who agreed to participate in the study.

MRI scans were performed in the SHIP center for clinical magnetic resonance research at the university of Greifswald using a whole-body 1.5 Tesla MR system (Magnetom Avanto; Siemens Medical Solutions, Erlangen, Germany). The protocol was identical for all participants and included axial T1-weighted ultra-fast gradient echo sequence (1.9/3.4 [repetition time ms/echo time ms]; flip angle 15°, 256 mm field of view, 1.0×1.0×1.0 mm voxel size and 176×256×176 acquisition matrix).

**Table 1:** Intra-Examiner Reproducibility – Mean Difference, Coefficients of Variability, and Intra-class Correlation statistics.

	First Reading	Second Reading	Second – First Reading	Coefficient of Variability		Intra-class Correlation	
Coordinate	Mean (SD)	Mean (SD)	Mean (SD)	[%]		Coefficient (95% CI)	
Left porion							
x	59.82 (4.85)	59.29 (4.78)	−0.52 (1.26)	1.27		0.961 (0.920–0.980)	
y	18.61 (6.72)	18.41 (6.84)	−0.20 (1.50)	4.46		0.976 (0.956–0.987)	
z	132.83 (12.21)	132.22 (12.08)	−0.61 (1.31)	0.56		0.993 (0.984–0.997)	
Right porion							
x	64.10 (4.91)	63.25 (5.02)	−0.86 (1.43)	1.40		0.945 (0.853–0.975)	
y	17.39 (7.46)	16.70 (7.59)	−0.69 (1.13)	5.86		0.985 (0.955–0.993)	
z	134.57 (11.87)	133.67 (11.87)	−0.90 (1.64)	0.75		0.988 (0.968–0.994)	
Left Orbitale							
x	34.08 (6.46)	33.99 (6.66)	−0.09 (0.97)		1.17		0.989 (0.989–0.994)
y	94.08 (8.53)	93.78 (8.62)	−0.30 (0.75)		0.50		0.996 (0.991–0.998)
z	134.43 (15.38)	134.17 (15.15)	−0.26 (0.91)		0.35		0.998 (0.996–0.999)

### (b) Landmark Detection

For data analysis, an open source dicom viewer (OsiriX v3.8.1) was used on two workstations with 27 inch monitors (iMac Quad core i7; Apple Corp. Cupertino, CA, USA). 3D coordinates for each image were calculated from the DICOM headers which were based on the MRI scanner coordinates. Osirix determined the coordinates (x, y, z) for each voxel and converted the actual calculated size of voxels to millimeters.

**Table 2:** Intra- and Inter-reader Reliability – Reader Differences in Distances for Points Defining FH Plane.

	Intra-Reader Reliability(Distance d to First Reading)	Inter-Reader Reliability (Distance d to First Reading of Reader 1 as the Gold Standard)			
	Reader 1	Reader 2	Reader 3	Reader 4	Reader 5
Left Porion					
Mean (SD), mm	2.0 (1.4)	2.3 (1.9)	2.2 (2.0)	2.1 (1.6)	2.2 (2.0)
≤1 mm, %	18.6	18.6	23.3	20.9	25.6
≤2 mm, %	62.8	55.8	69.8	67.4	62.8
≤3 mm, %	79.1	83.7	81.4	81.4	79.1
≤4 mm, %	86.0	90.7	81.4	88.4	83.7
≤5 mm, %	93.0	90.7	88.4	90.7	90.7
Right Porion					
Mean (SD), mm	2.4 (1.5)	2.9 (2.1)	2.3 (1.7)	2.0 (1.2)	2.2 (1.4)
≤1 mm, %	14.0	9.3	9.3	18.6	16.3
≤2 mm, %	46.5	41.9	53.5	58.1	53.5
≤3 mm, %	67.4	65.1	76.7	81.4	72.1
≤4 mm, %	83.7	76.7	88.4	90.7	90.7
≤5 mm, %	95.4	88.4	93.0	97.7	95.4
Left Orbitale					
Mean (SD), mm	1.3 (0.9)	1.9 (1.0)	1.7 (1.0)	1.6 (0.8)	1.3 (0.8)
≤1 mm, %	37.2	16.3	25.6	20.9	30.2
≤2 mm, %	79.1	62.8	69.8	69.8	79.1
≤3 mm, %	97.7	83.7	90.7	95.4	97.7
≤4 mm, %	97.7	95.4	95.4	97.7	100.0
≤5 mm, %	100.0	97.7	100.0	100.0	100.0

In our MRi scans, the X-axis represented the right-left direction, the Y-axis and Z-axis corresponded to the posterior-anterior and superior-inferior directions respectively. This predetermined system of three axes is always the same when the same set of images is uploaded to the software. Since a selected point will give the same (x,y,z) value in any new rendered slice view, multi-planar reconstruction (MPR) was used to accurately identify the three landmarks that define FH plane. Sagittal, axial, and coronal rendered slices, as well as the 3D image reconstruction were used to determine the 3D positional coordinates of Left and right porion (Po) and left orbitale (Or) based on their anatomical position. (Fig. 1 & 2).

**Table 3:** Intra- Examiner Reproducibility and Inter-Examiner Reliability – Reader Differences in Dihedral Angle of FH Plane.

	Intra-Examiner Reproducibility(Angle to First Reading)	Inter-Examiner Reliability (Angle to First Reading of Examiner)			
	Reader 1	Reader 2	Reader 3	Reader 4	Reader 5
Mean (SD), °	1.0 (0.7)	1.5 (0.8)	1.3 (1.0)	1.1 (1.0)	1.1 (0.8)
≤1°, %	53.5	25.6	53.5	58.1	55.8
≤2°, %	88.4	74.4	86.0	86.0	86.0
≤3°, %	97.7	93.0	90.7	88.4	97.7
≤4°, %	100.0	100.0	97.7	100.0	100.0
≤5°, %	100.0	100.0	100.0	100.0	100.0

The color coded locaters in both coronal and axial view ports were used simultaneously to detect the most inferior point on the infraorbital rim, orbitale landmark was then located on the sagittal view port. In the same manner, the locater in the coronal view port was used to outline the soft tissue and bone above the external auditory meatus, porion landmark was then located on the corresponding sagittal view port as the most lateral point in a low signal intensity area.

Furthermore, the color-coded axis locaters on the three planar views were used for further view angle adjustments to locate FH accurately (Fig. 3).

### (c) Evaluation of Reproducibility

Landmark coordinates for each image set were obtained by the main examiner two times in different sessions, and 1 time by 4 other examiners over a period of two weeks each, one week apart. All examiners were dentists and were previously trained in the use of Osirix software and craniofacial landmark identification. For investigator blinding, the images were identified by code and analyzed anonymously in random order.

**Figure 4:**
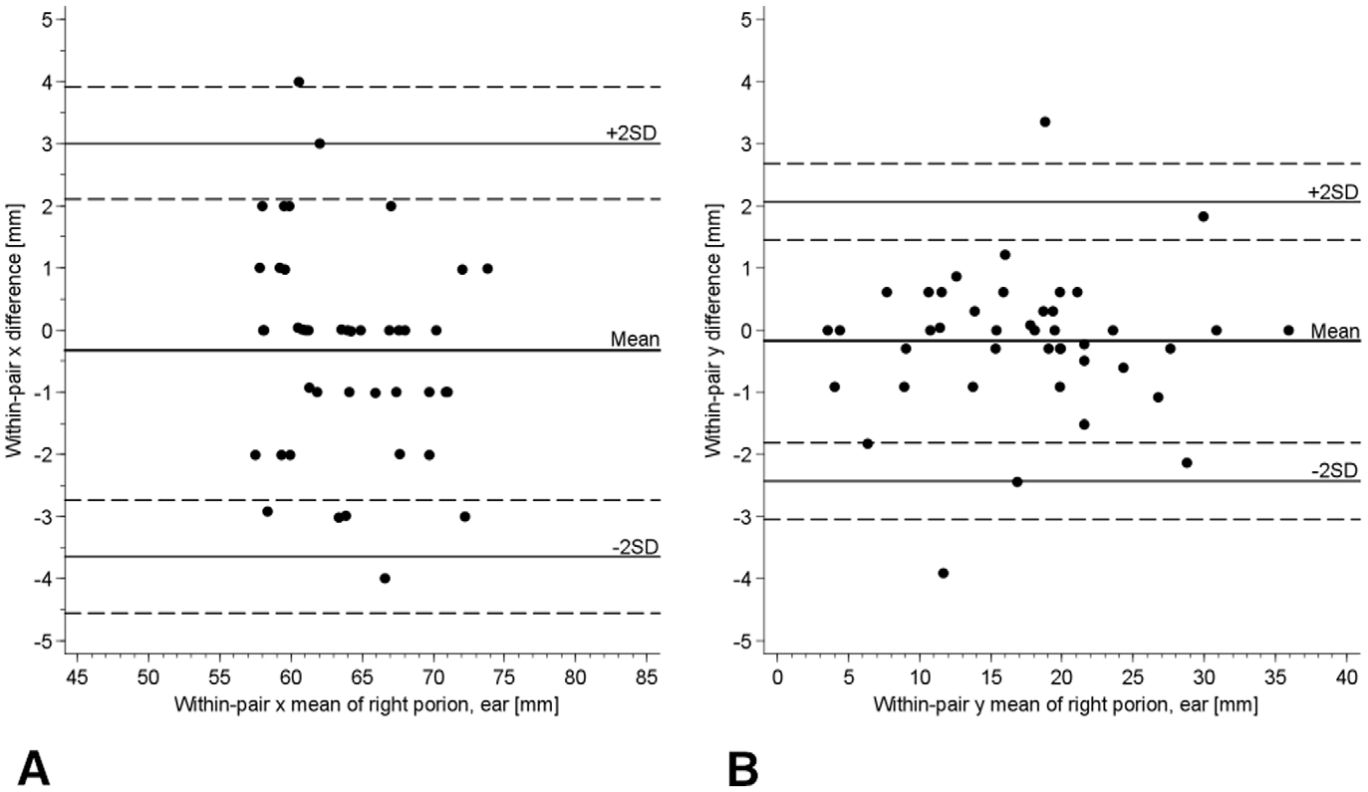
Bland-Altman Plot for Examiner 1 vs. Examiner 5. a) In x coordinate of right Porion. The horizontal lines represent the mean within-pair difference (−0.32 for examiner 5– examiner 1), the mean ±1.96 standard deviations (1 SD = 1.70), and the mean ±1.96 SD ± square root (3*SD^2^/n) for 95% CIs of limits of mean ±1.96 standard deviations. b) In y coordinate of right Porion.

Intra-examiner Reproducibility was assessed by using intraclass correlation coefficients (ICC) for the main examiner measurements. ICC was also used to calculate inter-examiner reliability by comparing the main examiner mean trial with the measurements of the other 4 examiners. Following the recommendation by Shrout & Fleiss [31], ICC (2,1) was used to assess if the standardized reading procedure can be effectively used by a variety of readers.

**Table 4:** Inter-Examiner Reliability – Mean differences, Coefficients of Variability (CV) and Intra-class Correlation statistics.

	Reader 1	Reader 2		Reader 3		Reader 4		Reader 5		Intra-class Correlation for 5 Examinerss
Coordinate	Mean (SD)	Difference to First Examiner Mean (SD)	CV [%]	Difference to First Examiner Mean (SD)	CV [%]	Difference to First Examiner Mean (SD)	CV [%]	Difference to First Examiner Mean(SD)	CV [%]	Coefficient (95% CI)
Left porion										
x	59.82 (4.85)	0.43 (1.64)	1.40	0.14 (2.02)	1.45	0.31 (1.39)	1.25	0.08 (1.83)	1.50	0.932 (0.897–0.959)
y	18.61 (6.72)	0.20 (1.82)	5.68	0.28 (1.66)	4.29	0.42 (1.61)	4.43	0.03 (1.60)	5.77	0.971 (0.955–0.982)
z	132.83 (12.21)	0.83 (1.35)	0.66	0.20 (1.51)	0.64	0.20 (1.54)	0.66	0.67 (1.63)	0.65	0.992 (0.987–0.995)
Right porion										
x	64.10 (4.91)	0.97 (2.34)	1.76	0.72 (1.95)	1.60	0.27 (1.33)	0.99	0.32 (1.70)	1.40	0.914 (0.871–0.948)
y	17.39 (7.46)	0.51 (1.16)	5.59	0.18 (1.31)	3.78	0.13 (1.01)	3.07	0.18 (1.15)	3.78	0.988 (0.981–0.993)
z	134.57 (11.87)	1.52 (1.56)	0.92	0.78 (1.29)	0.65	0.73 (1.44)	0.66	0.77 (1.41)	0.65	0.991 (0.984–0.995)
Left Orbitale										
x	34.08 (6.46)	0.07 (1.24)	1.95	0.26 (0.95)	1.32	0.13 (0.81)	1.08	0.23 (0.72)	0.96	0.987 (0.980–0.992)
y	94.08 (8.53)	0.66 (1.19)	0.83	0.32 (1.21)	0.72	0.03 (0.91)	0.51	0.03 (0.75)	0.46	0.990 (0.984–0.994)
z	134.43 (15.38)	0.18 (1.20)	0.47	0.67 (0.98)	0.47	0.81 (1.06)	0.57	0.52 (1.02)	0.46	0.997 (0.994–0.998)

Additionally, paired mean difference (D), standard deviation (SD), coefficient of variability and Bland-Altman plots were used as described and recommended by Szklo & Nieto. [32] The analyses and plots were performed using STATA/SE software, version 12.1 (StataCorp LP, College Station, Tex.).

In Bland-Altman plots, 95% confidence intervals (CI) of D–2S and D+2S were additionally drawn because only narrow 95% confidence interval reflect an appropriate sample size to detect reader differences. [33] Sample size calculation for these CIs in Bland-Altman plots are not available, therefore, we focused on the desired precision of ICC. The desired lower limit of 0.85 for ICCs of 0.90, 0.92, and 0.94 (interval widths of 0.10, 0.14, and 0.18, respectively) for 5 readers using the approximation given by Bonett [34] requires 36, 12, and 5 subjects, respectively. However, we decided to aim for 43 subjects because the interest is in the reliability of the plane rather than in those of a single coordinate.

**Figure 5:**
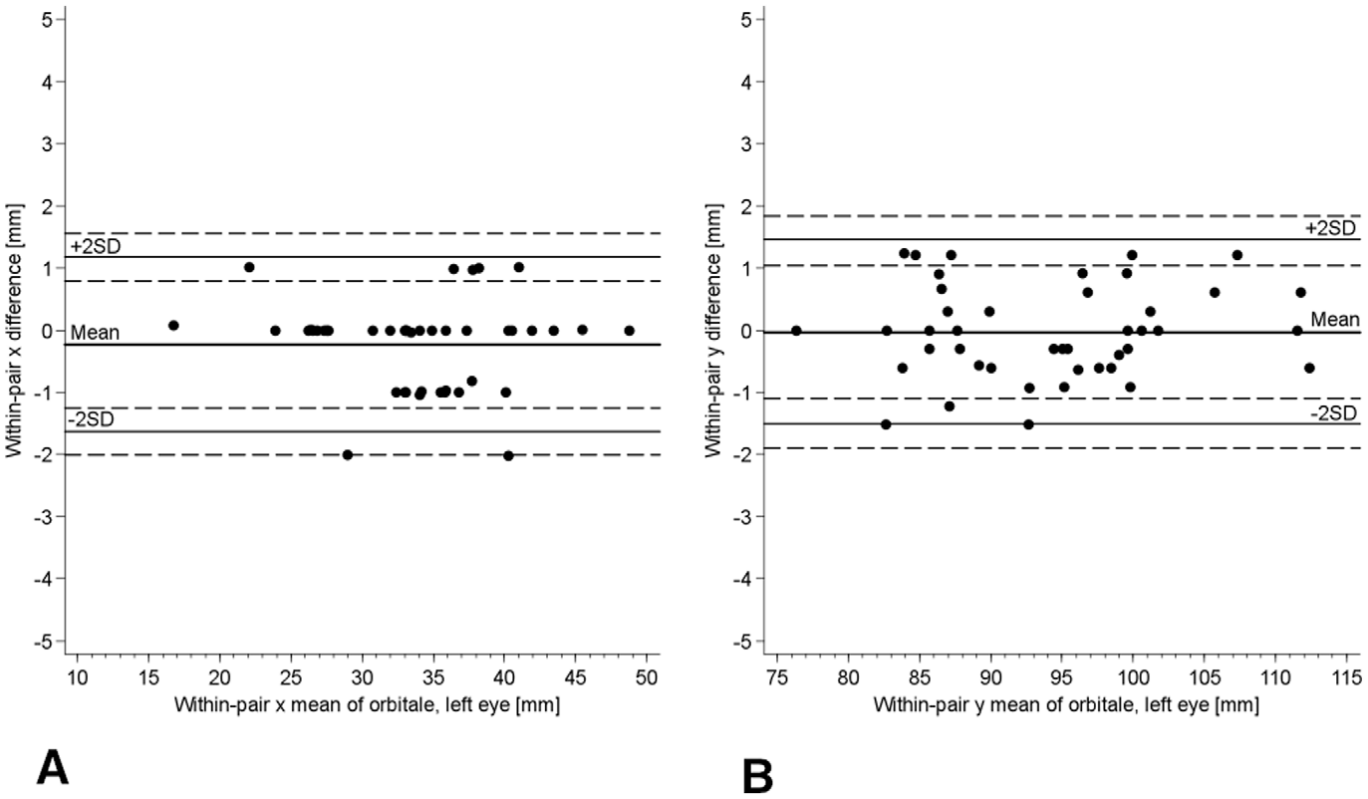
Bland-Altman Plot for Examiner 1 vs. Examiner 5. a) in x coordinate of left Orbitale (the shown digit preference reflects the slice thickness of 1 mm) b) In y coordinate of left Orbitale.

To assess the reliability of the plane in terms of distance, the spherical distance d between two readings of the same point was calculated (square root from (x1– x2)^2^ + (y1– y2)^2^ + (z1– z2)^2^ with indices for the two readings). To assess the reliability of the plane in terms of angulation, the dihedral angle between the planes from two readings was calculated.

## Results

The intra-examiner Reproducibility for each coordinate was greater than 0.94 in terms of ICC (Table 1). The 95% CIs were small with a lower limit of 0.85 indicating an excellent Reproducibility. The coefficients of variability were fairly low. The absolute systematic error (mean difference between both readings) for each Cartesian coordinate was lower than 1 mm. Systematic bias other than absolute error, such as proportional error, was graphically examined using Bland-Altman plots (not shown for intra-examiner Reproducibility). Intra-examiner reproducibility of the three landmarks in terms of distance showed mean differences between 1.3 to 2.4 mm (Table 2). Intra-examiner difference in the dihedral angle of FH was less than 3° for 97.7% of the readings with a mean of 1° (Table 3).

**Figure 6:**
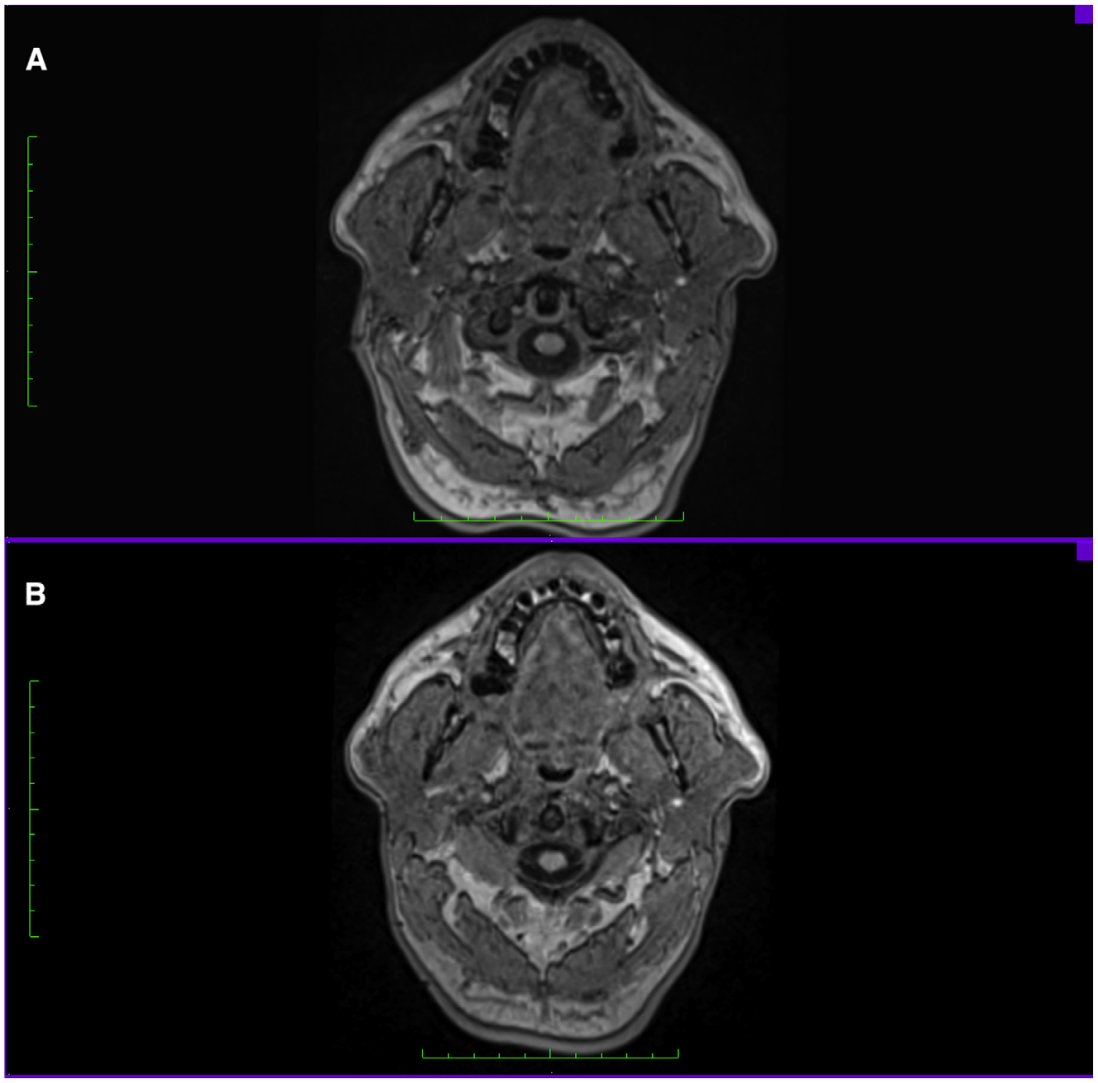
Axial head view, A. FH plane estimated prior scan on subjects head B. FH plane identified by landmarks on Images. (Note the differences in the muscles cross sectional area between both views).

The inter-examiner reliability for each coordinate was greater than 0.90 in terms of ICC (Table 4). The 95% CIs were small with a lower limit of 0.90, indicating an excellent reliability. The coefficients of variability were fairly low. The absolute systematic error (mean difference to the first examiner) for each Cartesian coordinate in the three points was lower than 1.52 mm. Bland-Altman plots showed no conspicuous pattern except for the expected digit preference in the x coordinate (Fig. 4a & 5a). This digit preference for a whole number reflects clearly the slice thickness of 1 mm and was absent in y and z coordinates (Fig. 4b & 5b). The 95% CIs (dashed lines) for the lines of ±2 SD (solid lines) were small, reflecting the sufficient sample size and the relatively small variation of the differences. Inter-examiner reliability of the three landmarks in terms of distance showed mean differences between 1.3 to 2.9 mm (Table 2). Differences in the dihedral angle between each examiner and the first examiner readings of FH was less than 3° for 88.4% of the readings with mean differences between 1.1° to 1.5° (Table 3).

## Discussion

Although FH plane is mentioned extensively in the literature as a reference plane for measuring craniofacial structures in MRI [35]–[38], no data have been published on its reproducibility. In this study, we evaluated the Intra- and Inter-examiner reproducibility of FH plane In MRI. We determined that this is important prior to its selection as a reference for cranial measurements, particularly in longitudinal studies that provide normative data for various craniofacial structures where any misjudgment in the reference plane may lead to false conclusions.

In the literature, many authors applied different methods to estimate FH inclination in relation to head posture. Machine ear rods, human skulls with metal markers were used to assist in its detection in cephalograms and CT. [11], [39]–[42] Pancherz et al [43], pointed out the high registration errors when a machine ear rod was used to identify porion point. They showed that ear rod markers gives Porion a soft tissue position (Po-m), which is unsuitable as a representation of the anatomical Porion (Po-a). It was concluded that the different locations of the two Porions will affect the angulation of FH and the measurements related to this reference line, subsequently, gross errors in the diagnosis and the treatment planning of orthodontic and surgical cases may result. In concordance with Pancherz et al study, Ludlow et al [44] reported high examiner variability in porion identification in conventional cephalograms and CT. They mentioned that identifying the mechanical location of porion could place this landmark more than 1 cm from its true anatomic position.

Photographs presented another approach of FH estimation accuracy [45], however, it was not possible to register the FH plane with a high degree of confidence on photographs, because this approach did not present the true clinical situation and the orbital rim could not be palpated and marked. Halazonetis et al [45] reported that direct comparison between the inclination of FH plane as measured from photographs or cephalometric radiographs was not valid.

In studies focusing on measuring craniofacial structures, FH plane was either estimated prior to MRI scans on the subject head while the subjects were in supine position [37], [38], or it was estimated perpendicular to the floor prior to CT scans. [46], [47] This approach might result in estimation errors and affect the accuracy of FH detection and the subsequent analysis, mainly because a simple head rotation would be enough to disturb the planned position of the head, making it difficult to maintain a horizontal plane angulation during scanning. Another shortcoming of FH estimation on subjects is the difference between the palpable landmarks and real landmarks on images. It was observed that the palpable soft tissue Frankfort plane (tragus-orbitale) was not parallel to the hard tissue Frankfort plane (porion-orbitale) and that the 2 planes show a deviation of 6° on average. [39] Our study was based on this consideration, since the majority of studies advocate the use of FH as a reference plane without questioning the differences between its detection on subjects heads and images and the consequent influence it carries on measuring craniofacial structures later on images. (Fig. 6).

Olszewski et al [10] studied the reproducibility of craniofacial landmarks and classified it into 4 groups based on the literature and the results of their study in CT. In their study, Porion was classified under mean inter-examiner reproducibility and Orbitale with low inter-examiner reproducibility. Later, in a study conducted by Lagravere et al [48], reproducibility of landmarks in Cephalograms and CT images was further assessed, in cephalograms, porion had moderate intra-examiner reliabilty for the y-axis (0.81) and mild inter-examiner reliability for the y-axis (0.46). In CT, Lagravere et al reported that right and left Porion showed the highest Intra-examiner mean differences in the X axis (2.62 and 3.37 mm, respectively) and high inter-examiner differences in the X axis in orbitale right and left (3.25 and 2.57 mm, respectively) and porion right and left (2.7 and 2.94 mm, respectively). Although differences in imaging modules, techniques and measurement methods make direct comparison of results reported in the literature on FH landmarks reproducibility rather unreliable, a general estimation on the 3D complexity of these landmarks can be concluded.

The results of our investigation showed that the examiner variability in detecting Porion (R/L) was slightly larger in the Sagittal plane than in the Axial and coronal planes. This observation is in accordance with that from other investigations [13], [44], [48] and it demonstrates the Medio-Lateral complexity of Porion in the MPR view due to its location on a widely curved bone. According to Ludlow et al [44] this variability in identifying porion is probably related to the inadequate definition of this landmark in the third dimension, they noted that while some examiners localized porion in the soft tissues of the ear canal, others localized it on the bone/soft tissue margin.

We attempted in this study to measure the variation in FH landmarks detection and the effect it carries on FH plane angulation. Differences in the 3D location of one of the landmarks caused up to 3° deviation of FH plane between examiners. Since variation in each of the three axes of a landmark will not contribute equally to its location, it may be difficult to establish which landmark is the primary contributor to the variability of FH plane angulation.

Our study found excellent inter- and intra-examiner reproducibility for the three points studied with 5 examiners utilizing 3D MRI images with 3 coordinate values for each point and using the anatomical definition for each landmark. Our study focused on the examiner reliability of FH detection on MRI, we did not intend to address specific measurement errors that may result when FH is not detected directly on MRI images. Future studies need to demonstrate the differences in specific craniofacial measurements when FH is estimated directly on subjects and when it is defined on images.

### Conclusion

This study revealed excellent intra-examiner and inter-examiner reproducibility of Frankfort Horizontal plane through 3D landmark identification in MRI using freely available software Osirix. Sufficiently stable landmark-based reference plane could be used for different treatments and studies.
