# Correction: Reproducibility of Frankfort Horizontal Plane on 3D Multi-Planar Reconstructed MR Images

**DOI:** 10.1371/annotation/6bc54ac4-3639-447d-92bc-dad0ddb24c1e

**Published:** 2012-11-08

**Authors:** Amro Daboul, Christian Schwahn, Grit Schaffner, Silvia Soehnel, Stefanie Samietz, Ahmad Aljaghsi, Mohamad Habes, Katrin Hegenscheid, Ralf Puls, Thomas Klinke, Reiner Biffar

The name of the seventh author is spelled incorrectly. The correct author name is: Mohamad Habes. 

